# Discontinuation of performance-based financing in primary health care: impact on family planning and maternal and child health

**DOI:** 10.1007/s10754-022-09333-w

**Published:** 2022-05-18

**Authors:** Amira El-Shal, Patricia Cubi-Molla, Mireia Jofre-Bonet

**Affiliations:** 1 Department of Economics, Faculty of Economics and Political Science, Cairo University, 12613 Giza, Egypt; 2 Office of Health Economics, SW1E 6QT London, UK; 3 Department of Economics, City, University of London, EC1V 0HB London, UK

**Keywords:** Performance-based financing, Performance-based pay, Maternal health, Child health, Egypt, SDG3, D9, I11, I18

## Abstract

Performance-based financing (PBF) is advocated as an effective means to improve the quality of care by changing healthcare providers’ behavior. However, there is limited evidence on its effectiveness in low- and middle-income countries and on its implementation in primary care settings. Evidence on the effect of *discontinuing* PBF is even more limited than that of introducing PBF schemes. We estimate the effects of discontinuing PBF in Egypt on family planning, maternal health, and child health outcomes. We use a difference-in-differences (DiD) model with fixed effects, exploiting a unique dataset of six waves of *spatially* constructed facility-level health outcomes. We find that discontinuing performance-based incentives to providers had a negative effect on the knowledge of contraceptive methods, iron supplementation during pregnancy, the prevalence of childhood acute respiratory infection, and, more importantly, under-five child mortality, all of which were *indirectly* targeted by the PBF scheme. No significant effects are reported for directly targeted outcomes. Our findings suggest that PBF can induce permanent changes in providers’ behavior, but this may come at the expense of *non-contracted* outcomes.

## Introduction

In 1997, the Government of Egypt (GoE) launched a large-scale public health sector reform program (HSRP), which came into operation in 2000. Later in 2001, a performance-based financing (PBF) scheme was integrated into the Program. According to this scheme, governorate-level financial intermediaries, known as the Family Health Funds (FHFs), became entitled to pay monthly incentives to healthcare providers in “contracted” healthcare facilities based on pre-specified performance criteria. To qualify for financial incentives, facilities were required to meet pre-determined standards in a set of 11 indicators that covered several aspects of service provision, both curative and preventive. In 2008, however, the financial incentives were discontinued due to the financial unsustainability of the FHFs. The PBF scheme was replaced by case-based reimbursement.

Financial incentives to healthcare providers are advocated as an effective means to alter their behavior, with the aim of improving quality of care. While there is considerable enthusiasm for PBF, there is little rigorous evidence on its effectiveness in low- and middle-income country (LMIC) settings, especially if implemented at scale (Paul et al., 2018; Das et al., 2016; Witter et al., 2012; Eldridge & Palmer, 2009). Moreover, previous research focused on processes and outputs, such as health service coverage and utilization, instead of outcomes and impact. Where evidence is available, it is inconclusive (James et al., 2020). Some studies argue that, when implemented in LMICs, PBF does not improve the performance of health systems (e.g., Paul et al., 2018). However, a systematic review of the literature by James et al. (2020) indicates several positive effects of results-based financing (RBF) on maternal, newborn, and child health in LMICs.

To date, very few studies explored the effects of *discontinuing* rather than *introducing* provider incentives, particularly within the health system of an LMIC. We could only identify one study in an LMIC (in the Democratic Republic of Congo), showing that the motivation of workers in health facilities where PBF was removed is lower than those who never received incentives (Maini et al., 2019).

Studies that examined the effects of removing performance-based incentives in high-income countries provide mixed results. Some studies found that performance improvements were generally sustained after discontinuing the incentives (e.g., Kontopantelis et al., 2014). Other studies show that removing the incentives resulted in an immediate decline in the performance of quality-of-care measures (e.g., Minchin et al., 2018). In parallel, conflicting results are observed concerning the effects of discontinuing performance-based incentives on healthcare expenditure and cost containment (Fiorentini et al., 2013; Dusheiko et al., 2006).

In general, earlier research focused on structure indicators (such as equipment or personnel) or process indicators (such as treatment plan or diagnosis) rather than patient outcomes. To our knowledge, no one has applied the analysis to patient outcomes in LMICs. Since many PBF schemes have been in operation for several years, the need to improve policy design requires an insight into the effects of discontinuing these incentives either partially or totally. Such discontinuation may be fueled by changes in policy priorities, the ineffectiveness of some of the schemes, or a consequence of other coetaneous schemes.

This study investigates the effect of discontinuing provider incentives in Egypt on the primary care areas of family planning, maternal health, and child health. We explore the effect of PBF discontinuation on two types of health outcomes: those *directly* targeted by the PBF scheme (which we refer to as “contracted” outcomes) and those not directly linked to the scheme (“non-contracted” outcomes). Data is obtained from six waves (1992,1995, 2000, 2005, 2008, and 2014) of the Egypt Demographic and Health Survey (DHS). We *spatially* link women interviewed in each of the six waves to their nearest mapped health facilities using the Global Positioning System (GPS) coordinates of the women’s home address and the facilities. The spatial mapping allows us to combine facility-level health outcomes in a panel and estimate the effects of discontinuing PBF at the facility level. We employ a difference-in-differences (DiD) model with fixed effects.

Many LMICs developed and integrated PBF models into their health systems to increase the use and quality of healthcare services. Some governments have recently considered discontinuing these models to secure fiscal space in the context of COVID-19. Therefore, as the available evidence on the causal effects of removing or replacing performance-based provider incentives is weak, our study is timely.

Our results show that PBF discontinuation negatively affected the knowledge of contraceptive methods (-11.3% points or ppts), the likelihood of receiving iron supplements during pregnancy (-9.4 ppts), the prevalence of childhood acute respiratory infection or ARI (5.5 ppts), and, more importantly, under-five child mortality (2.7 ppts). All these outcomes were non-contracted. No effects are detected for contracted outcomes. We demonstrate that although the behavior of healthcare providers can be favorably modified to serve national health sector goals permanently through tying performance to financial incentives, a sudden scheme discontinuation may result in a negative impact on key health outcomes.

## Literature review

A systematic review of the literature identified nine relevant studies on the effect of discontinuing provider incentives. Only one of these studies was conducted in an LMIC, specifically the Democratic Republic of Congo, and indicated that in health facilities where PBF was removed, the motivation of workers was lower than the motivation of those who never received incentives (Maini et al., 2019).

Six studies investigated the effect of discontinuing incentives on recorded quality-of-care measures. Benzer et al. (2014) found that performance improvements that occurred in Veterans Health Administration (VA) medical centers in the United States for three common conditions (acute coronary syndrome, heart failure, and pneumonia) were sustained for up to three years after performance-based incentives were discontinued. These sustained improvements – and the associated higher quality of care – may reflect the permanent adoption of new standards of care driven by PBF.

Hysong et al. (2011) also investigated the discontinuation of incentives within the VA. They used outpatient clinical performance measure data from VA’s External Peer Review Program in the United States to examine the stability of performance after changing status from being actively monitored (i.e., incentivized) to being passively monitored (i.e., no incentive) and vice versa. The study found that all quality-of-care measures remained stable or improved over time regardless of whether or not a measure was incentivized. Quality did not deteriorate for any of the measures for which incentives were removed.

Similarly, Boland et al. (2010) found that the significant positive effect on quality of care resulting from a radiologist PBF program in the United States, measured by expediting final report turnaround times, persisted after discontinuation of the program.

In the United Kingdom, Kontopantelis et al. (2014) explored the effect of discontinuing financial incentives for some aspects of care for patients with asthma, coronary heart disease, diabetes, stroke and psychosis on eight clinical quality indicators withdrawn from a national incentive scheme. They found that the level of performance achieved prior to the incentive discontinuation was generally maintained, with some differences observed by indicator and disease condition.

Lester et al. (2010) found that discontinuing incentives was associated with a decrease in screening for diabetic retinopathy and screening for cervical cancer in the United States. More recently, Minchin et al. (2018) also showed that removing financial incentives in the United Kingdom for primary health practices resulted in an immediate decline in the performance of quality-of-care measures.

Two studies examined the effect of discontinuation on healthcare expenditure and cost containment. Fiorentini et al. (2013) estimated the effect of discontinuing financial incentives offered to primary healthcare (PHC) providers in exchange for containing hospital expenditure in an Italian region and found no effects on avoidable hospital expenditure and total hospital expenditure. Moreover, they indicated that discontinuation of incentives did not affect physicians’ behavior. However, an earlier study by Dusheiko et al. (2006) showed that abolishing a fundholding scheme^1^ in the United Kingdom increased former fundholders’ admission rates for chargeable elective admissions. The study also found that the effect on the early wave fundholders was stronger than on later wave fundholders.

The reviewed studies showed conflicting results, but most provided evidence that the quality of care does not always deteriorate for the measures for which incentives were removed. Conflicting results were also observed for healthcare expenditure and cost containment.

We additionally reviewed the evidence on the effects of introducing provider incentives to anticipate the likely effects of discontinuing these incentives in LMICs (see Appendix A). Overall, evidence on the effectiveness of introducing PBF on improving family planning, maternal health, and child health outcomes is mixed. This is partly because PBF schemes introduced in LMICs are not identical. The most consistent evidence of the effectiveness of PBF is for the quality of antenatal care (ANC) and medical treatment among children. These are the areas that we expect to be affected the most if provider incentives are discontinued.

## Performance-based financing in Egypt

The providers of PHC in Egypt are the Ministry of Health of Population (MoHP) (public), the Health Insurance Organization (HIO)^2^ (public), and the private sector. The MoHP operates a nationwide network of PHC facilities, which serve as the “insurer of last resort” by offering free or substantially subsidized PHC services to the uninsured population.

In the early 1990s, the main focus of Egypt’s national health strategy was to achieve universal health coverage (UHC). The GoE pressured the HIO, Egypt’s largest health insurer, to rapidly expand coverage to new groups, including infants and school children. The pressing need to achieve rapid progress towards UHC did not consider the appropriate health system financing required to ensure sustainability and the quality of healthcare services. Over 60 per cent of all PHC visits took place in private facilities despite the massive capacity, low cost, and physical availability of public PHC facilities across the country. The underutilization of PHC services was primarily induced by the poor quality of care provided. Private out-of-pocket (OOP) expenditures were also substantial (World Bank, 2004). In addition, the long-term financial stability of the health system was threatened by allocative and technical inefficiencies, especially considering the expanding insurance coverage and high population growth.

The GoE launched a large-scale HSRP in 1997 to address these fundamental challenges to the health system. The Program’s objective was to provide a package of essential healthcare services, referred to as the Basic Benefit Package (BBP), for the whole population based on the five guiding principles of universality, quality, equity, efficiency, and sustainability.

**Fig. 1:**
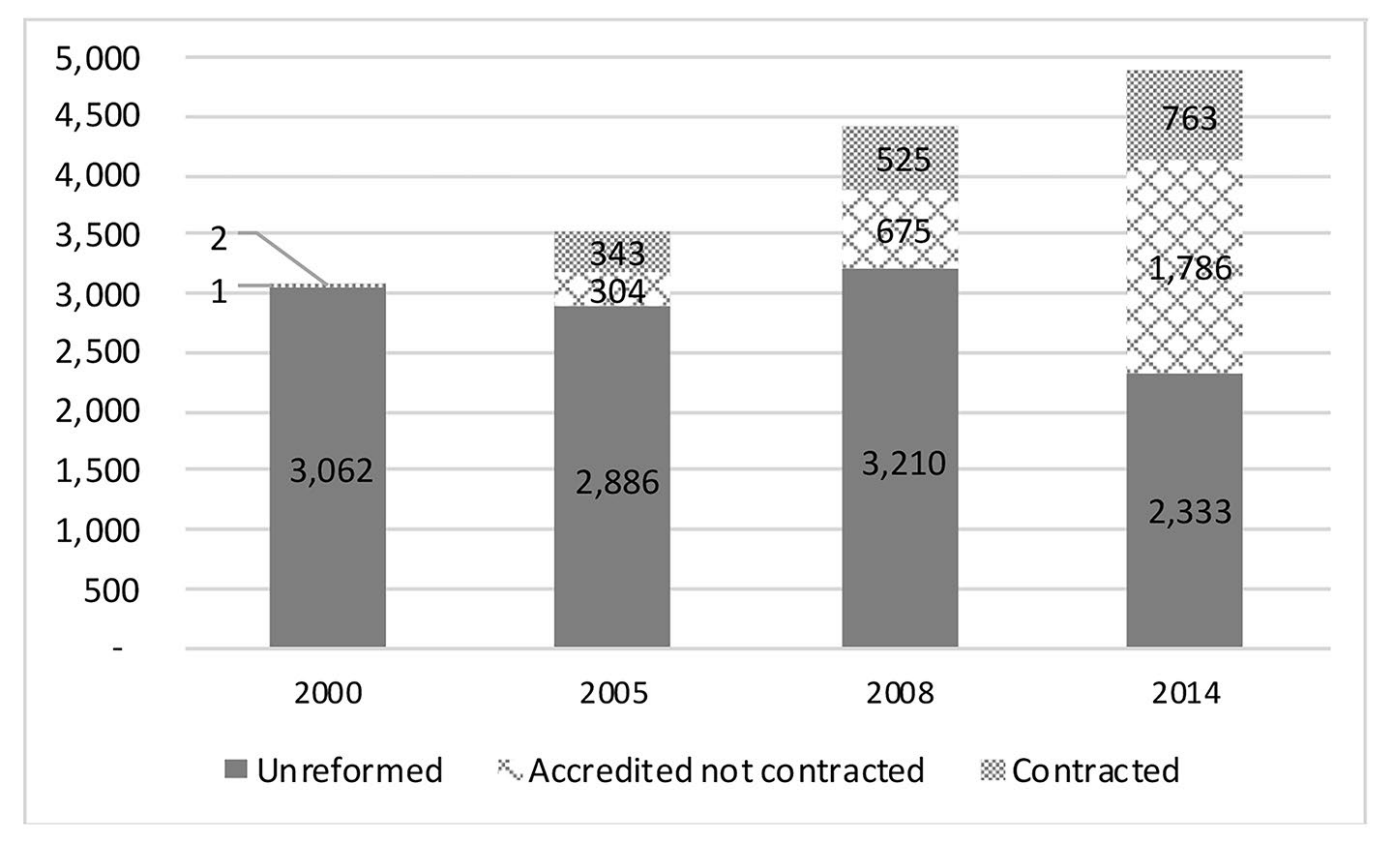
Health facilities in Egypt by reform status (2000–2014) Source: Our own calculations based on data from Egypt’s MoHP

In addition to introducing the BBP, the Program affected how PHC was financed, delivered, organized, and managed. The HSRP’s service delivery component involved three central supply-side interventions: renewal of the PHC infrastructure and equipment, human resource development, and quality assurance through a facility accreditation system. Health facilities subject to these interventions were referred to as “accredited” (El-Shal et al., 2021a). The Program’s financing component involved two interventions: the rechanneling of funds from direct financing to PBF of healthcare providers and, later in 2003, instituting a non-linear price system for the uninsured at the point of delivery (El-Shal et al., 2021b). Facilities participating in both the service delivery and financing components of the HSRP were referred to as “contracted”. The rest of the facilities remained “unreformed” (World Bank, 2004). We decompose the population of Egypt’s PHC facilities by reform status in Fig. 1.

Our intervention of interest concerns the PBF, to which only contracted health facilities had been exposed. Our treatment is defined as the discontinuation rather than the introduction of PBF. First, we zoom in on the design of the PBF scheme. This scheme was integrated with the HSRP in 2001 to increase Egypt’s quality and use of health services. Under this scheme, a governorate-level financial intermediary, the FHF^3^, paid monthly financial incentives against pre-defined performance criteria to healthcare providers in contracted facilities who delivered the BBP. The pre-determined standards included 11 indicators identified by the MoHP to reflect various aspects of service provision. The PBF scheme intended to address priority health concerns in Egypt reflected by these indicators, including maternal and child health, reproductive health/family planning, tuberculosis (TB), and immunization.

In Table 1, we provide a list of the 11 indicators and the required target for each indicator. The better a facility performed in these indicators, the higher the incentives paid to it by the FHF. Facilities achieving more than 75 per cent of the indicator targets received 100 per cent of the financial incentives. Those reaching 61–75 per cent received 50 per cent of the financial rewards, and those attaining less than 61 per cent none.

If a facility met specific targets, the relevant FHF would make a cash payment to the facility manager, who then distributed the incentives to the staff involved in achieving the target. All workers in contracted facilities were eligible to receive the financial incentives, including doctors, nurses, technicians, administrators, other health workers, and support staff. They received a base salary, typically low, plus an incentive payment up to 275 per cent of their base salary. To identify which staff participated in achieving the target, each facility had its own pre-determined protocol based on a point system. This system was, in turn, based on several variables, such as qualifications, experience, the number of days worked, and the effort exerted to achieve indicator targets in each area. The total payment made to a facility was divided by the sum of the points earned by staff and multiplied by the number of points for each worker. This calculation determined the amount of cash payment each worker received each month.

**Table 1:** Performance indicators of the PBF scheme

	Indicator	Target
1	Number of visits per day per physician	10–24
2	Number of drugs per visit	Less than 2
3	Rate of patient referral to the district hospital	1–8%
4	Rate of completion of visit encounter forms	More than 98%
5	Patient satisfaction rate	More than 90%
6	Rate of completion of medical records data	More than 90%
7	Family planning (Years of protection)*	More than 50%
8	Number of children fully vaccinated in the catchment area	95%
9	Patient waiting time	Less than 20 min
10	Number of ANC visits per pregnant woman	More than half a visit per month
11	Facility adherence to medical protocols	More than 98%

ANC: Antenatal care. *estimated years of protection provided by contraceptive methods based upon the volume of all contraceptives sold or distributed free of charge to beneficiaries during that period

*Source*: Egypt’s MoHP

In non-contracted facilities, staff incentives (“bonuses”) were not performance related. Instead, they were paid by the MoHP as a salary supplement as a top-off of their regular salary. A “de-facto” comparison of the two schemes indicates that if the performance of a contracted facility was higher than average, the incentives received by its staff surpassed those received in non-contracted facilities. However, at average performance levels, the incentives received at a contracted facility were more stringent than those at a non-contracted facility, making the latter more attractive for staff.

In order to afford paying monthly incentives to contracted providers, FHFs were financed through different sources, mainly revenues from the (then) new price system (roster fees, visit fees, and copayments); MoHP’s contributions on behalf of the uninsured; HIO’s reimbursements on behalf of its insured individuals using contracted PHC facilities; and official donations from internal and external agencies.

In general, selection into the HSRP – and the *PBF introduction* in particular – was not random but responded to a socio-economic vulnerability index score to target the most vulnerable populations.^4^ Targeting occurred at the district level, not at the village or facility levels. There was no concern that a woman might bypass her closest contracted PHC facility in favor of an unreformed one because she is obliged by the MoHP to use only the PHC facility in her catchment area (MoHP, 2004).

In September 2008, due to the financial unsustainability of the FHFs, the PBF scheme was discontinued at once in all contracted facilities. Therefore, selection into the *PBF discontinuation* was random. The PBF scheme was replaced by a case-based reimbursement scheme tied to the number of outpatient visits.

**Anticipated effect of PBF discontinuation.** Contract theory suggests that contracts can be designed to induce agents to perform or not perform actions (Koszegi, 2014). Incentive-based contracts address information asymmetries in provider-patient and payer-provider relationships. Since the principal usually cannot observe the effort of the agent and outcomes (which can be observed) depend on the agent’s efforts, contracts can be made conditional on these outcomes. The outcomes act as proxies of the agent’s effort.

The relationship between the MoHP and PHC providers in Egypt fits into a typical principal-agent framework in which a healthcare provider’s (agent’s) level of “effort” affects the quality of health services delivered, but the “effort” is unobservable to the payer (principal). So, the payer reimburses the provider according to contracted outcomes, not according to the unobservable choice of effort. Thus, we are considering a healthcare service contract problem here in which (i) the MoHP offers a contract to a PHC provider to deliver outpatient services contingent on observable health outcomes; (ii) the provider chooses the level of (unobservable) effort, which is costly to her/him; and (iii) the provider has a separable utility function that is increasing in compensation and decreasing in effort. In this context, a PHC provider chooses the level of effort that maximizes her/his expected utility given the cost associated with effort and the type of contract faced.^5^

If the financial incentives received from the FHF managed to induce a higher performance of PHC providers, we expect the discontinuation of the incentives to induce a decrease in effort and, thus, a deterioration in health outcomes. In this paper, we estimate the effect of PBF discontinuation on health outcomes due to the potential induced change in providers’ effort. We hypothesize that replacing the PBF scheme with case-based reimbursement might have had *direct* adverse effects on the quality and the use of health services initially targeted by the scheme (see Table 1) and *indirect* adverse effects on the quality and use of non-contracted PHC services. PBF, as a reimbursement mechanism, prioritizes the quality of care, whereas case-based reimbursement typically prioritizes health facility efficiency and healthcare cost control over the intensity and quality of care (Miller, 2009).

## Empirical strategy

We use a DiD model with fixed effects to estimate the effect of discontinuing performance-based provider incentives at contracted health facilities across Egypt. The model feeds on a unique panel dataset of six waves of constructed facility-level health outcomes and information on the reform status of each PHC facility in the country, covering the period from 1992 to 2014. Two different interventions were introduced during this interval: (1) contracting PHC facilities and (2) discontinuing PBF of healthcare providers. Contracting PHC facilities (and therefore the introduction of PBF) was staggered, with different facilities joining at different times post 2000. The change of PBF to case-based reimbursement happened all at once in all contracted facilities in 2008. We consider *PBF discontinuation* as our “treatment” of interest. As the 2008 DHS wave data were collected *before* the PBF discontinuation, we set 2014 as the post-treatment year.

We include unreformed and contracted facilities in the analysis regardless of the date of contracting. By definition, only contracted facilities were subject to PBF discontinuation (i.e., subject to “treatment”). Thus, its effect is estimated relative to facilities that remained unreformed (i.e., untreated or controls), or in other words, never provided financial incentives to providers.

For each health facility *i* at time *t*, we estimate the following model for each health outcome:

$${HO}_{it}=\alpha + \beta {CONT}_{it}+\gamma {PBF\_DISCONT}_{it}+{\theta }_{i}+{\lambda }_{t}+{\epsilon}_{it}$$

*HO*_*it*_ denotes the health outcome of interest (i.e., family planning, maternal health, or child health outcome) of facility *i* in year *t*. *CONT*_*it*_ equals 1 if facility *i* is contracted in year *t*, and *0* otherwise. *PBF_DISCONT* takes value 1 if PBF is no longer in operation in a formerly contracted facility *i* in year *t* (i.e., in 2014), and *0* if not. *θ*_*i*_ and *θ*_*i*_ are sets of facility and year fixed effects, respectively. *λ*_*t*_ s the facility effect that captures all time-invariant factors correlated with the outcome. *λ*_*t*_ is the year effect that captures common secular trends. The error term, *ε*_*it*_, captures the residual variation. It should be noted that for a health outcome like modern contraceptive prevalence *(mcp)*, a *positive* sign of the coefficients *β* or *γ* indicates a *positive* effect of treatment. In contrast, for a health outcome like under-five mortality *(childmort)*, a *negative* sign is interpreted as a *positive* effect.

Our model specification and estimation strategy attenuate endogeneity concerns at different levels. The facility fixed-effect term captures the unobserved facility-specific heterogeneity, reflecting that some facilities could have some characteristics that made them more likely to participate in the treatment and/or to benefit/not benefit from it (Wooldridge, 2002). The specification also accounts for the time trend, particularly relevant for capturing Egypt’s political and economic disruptions between 2008 and 2014. The intercept *α* absorbs the time-invariant characteristics.

Given the non-random allocation of the PBF scheme (introduction) across districts, our identification strategy relies on the validation of the parallel-trends assumption. Thus, before estimating Eq. (1), we verify that the difference between the health outcomes of contracted and non-contracted facilities would have remained on similar tracks in the absence of contracting.

We follow Mason et al. (2017) and obtain the pre-treatment slopes of health outcomes by regressing their change before 2005 on a dummy variable that denotes contracting “treatment” afterwards. Facilities that became contracted as of 2005 are defined as treated, and facilities that continued to be unreformed are the controls.

In Table 2, we present the estimated mean changes in the outcomes of both contracted (as of the year 2005) and non-contracted facilities drawing from 1992, 1995, 2000, and 2005 DHS waves. The “treatment” dummy estimates are not statistically significant for all reported outcomes, which indicates the absence of a pre-trend and validates the parallel-trend assumption, soothing selectivity-bias concerns regarding the PBF introduction.

**Table 2:** Estimated mean difference in health outcomes (1992–2005)

	Outcome	“Treatment” dummy
Family planning	Modern contraceptive prevalence	2.339
		(3.990)
	Knowledge of side effects of contraceptives	3.188
		(6.904)
	Knowledge of alternative contraceptives	16.081
		(12.597)
ANC	ANC by skilled health personnel	5.208
		(5.820)
	4 + visits	6.149
		(6.053)
	Iron supplementation	4.789
		(10.844)
Child health	ARI prevalence	-0.333
		(3.907)
	Fever prevalence	-1.949
		(4.906)
	Diarrhea prevalence	-2.802
		(3.765)
	Under-5 mortality	-2.771
		(1.848)

Each *row* represents a separate regression. Year dummies are included in all estimations. Standard errors are reported in parentheses. *, **, and *** denote statistical significance at the 10%, 5%, and 1% levels, respectively

## Data

**Health outcomes**. We use six waves of the Egypt DHS (1992, 1995, 2000, 2005, 2008, and 2014) to calculate measures of family planning, maternal health, and child health outcomes at the facility level (the level at which the HSRP interventions were introduced). The Egypt DHS consists of two questionnaires, one for households (HH) and another for ever-married women (EMW). In Appendix B, we summarize the sample selection, the survey coverage, and the response rate of the survey waves.

This study focuses on family planning, maternal health, and child health outcomes that reflect the health services targeted by the PBF scheme, whether directly or indirectly, and possibly the quality of these services. Table 3 lists the calculated outcomes and shows how they were linked to Egypt’s PBF scheme. We obtained the relevant data to calculate all the outcomes of interest from the EMW questionnaire, which provides information on the following topics: respondent’s background; reproduction; contraceptive knowledge and use; fertility preferences and attitudes about family planning; pregnancy and breastfeeding; child immunization and health; child nutrition; husband’s background, women’s work, and health care; female circumcision; and HIV/AIDS and other sexually transmitted infections.

**Table 3:** Description of health outcomes and their link to the PBF scheme

	Outcome	Description*	Relevant PBF scheme indicator	Link to scheme
Family planning	*mcp*	Percentage of women currently using any modern contraceptive method	Number of new users of all types of modern contraceptive methods among married women of reproductive age in the catchment area	Direct
	*contsid*	Percentage of current users of selected contraceptive methods informed of side effects of the method used	Number of new users of all types of modern contraceptive methods among married women of reproductive age in the catchment area	Indirect
	*contoth*	Percentage of current users of selected contraceptive methods informed of other methods of contraception that could be used	Number of new users of all types of modern contraceptive methods among married women of reproductive age in the catchment area	Indirect
ANC	*ancprov*	Percentage of women attended for ANC by skilled health personnel	Number of married and pregnant women receiving regular ANC visits compared to the total number of married and pregnant women in the catchment area	Direct
	*anc4*	Percentage of women who received four or more ANC visits	Number of married and pregnant women receiving regular ANC visits compared to the total number of married and pregnant women in the catchment area	Direct
	*anciron*	Percentage of women who received iron supplements as an ANC component	Number of married and pregnant women receiving regular ANC visits compared to the total number of married and pregnant women in the catchment area	Indirect
Child health	*childari*	Percentage of children under five years of age ill with a cough accompanied by short and rapid breathing at any time during the two weeks preceding the interview	Number of visits per day; rate of patient referral to the district hospital	Indirect
	*childfev*	Percentage of children under five years of age ill with a fever at any time during the two weeks preceding the interview	Number of visits per day; rate of patient referral to the district hospital	Indirect
	*childdiarr*	Percentage of children under five years of age ill with diarrhea at any time during the two weeks preceding the interview	Number of visits per day; rate of patient referral to the district hospital	Indirect
	*childmort*	Percentage of deaths at age 0–5 years to live-born children	Number of visits per day; rate of patient referral to the district hospital; number of children fully vaccinated in the catchment area	Indirect

*Definitions are obtained from the World Health Organization (WHO)

To construct facility-level health outcomes, we first associate each woman interviewed in each of the Egypt DHS waves to her nearest mapped facility based on the GPS coordinates of the interviewed women’s home address and the facilities. This allows us to identify women who live in the catchment area of contracted facilities and women in the catchment of non-contracted facilities. We use the software Quantum GIS 2.8.2 to perform the spatial join while exploiting all PHC facilities across Egypt. Second, health outcomes are computed at the facility level and combined in a panel. We rely on women’s self-reported data to construct health outcomes and not facility self-reported data that is suspected of being biased in either direction

**Treatment.** The analysis has two policy variables. The first reflects whether a PHC facility was contracted or not and thus exposed to quality assurance through facility accreditation, authorization to collect user fees and drug copayments from beneficiaries, and PBF of healthcare providers. Contracting (the introduction of PBF) was staggered due to the contractual agreements between PHC facilities and the FHFs taking effect at different times after the year 2000. The second policy variable reflects the 2008 discontinuation of PBF in PHC facilities (which is the treatment for our purposes) or, specifically, replacing the PBF scheme with case-based reimbursement. Our post-treatment year is 2014 (as mentioned, the 2008 DHS wave was collected *before* the PBF discontinuation)

We construct the two policy variables drawing from information from Egypt’s MoHP on the reform status of each PHC facility in the country during the 2000–2014 period

## Results

### Descriptive statistics

We conducted two-sample t-tests to test the difference between the (mean) health outcomes of treated (contracted) and control (non-contracted) PHC facilities in the baseline year (2005) and the post-treatment year (2014). Table 4 shows no significant differences in health outcomes, except for iron supplementation during pregnancy *(anciron)* (with treated facilities doing better at the baseline than their untreated counterparts) and the prevalence of childhood diarrhea *(childdiarr)* (worse results for facilities in the treated group after PBF discontinuation).

**Table 4:** Two-sample t-test of (mean) health outcomes of treated and control facilities

		Beginning of PBF operation (2005)				Post PBF discontinuation (2014)				Change from 2005 to 2014 in ppts	
Outcome		Control_05	Treated_05	Difference (T - C)	Treated better than control?	Control_14	Treated_14	Difference (T - C) (2)	Treated better than control? (2)	Control	Treated
Modern contraceptive prevalence	*(mcp)*	49.371	50.546	1.175	Better	50.566	53.290	2.724	Better	2%	5%
				(3.286)				(2.817)			
Knowledge of side effects of contraceptives	*(contsid)*	46.029	44.121	-1.908	Worse	44.808	42.887	-1.921	Worse	-3%	-3%
				(4.998)				(4.094)			
Knowledge of alternative contraceptives	*(contoth)*	51.714	54.091	2.377	Better	56.822	58.080	1.258	Better	10%	7%
				(4.626)				(4.058)			
ANC by skilled health personnel	*(ancprov)*	70.738	77.830	7.092	Better	90.473	90.973	0.501	Better	28%	17%
				(4.531)				(2.303)			
4 + ANC visits	*(anc4)*	59.733	63.148	3.415	Better	83.430	80.077	-3.353	Worse	40%	27%
				(4.904)				(2.854)			
Iron supplementation during pregnancy	*(anciron)*	48.809	64.198	15.389***	Better	66.727	71.853	5.126	Better	37%	12%
				(4.720)				(3.635)			
Childhood ARI prevalence	*(childari)*	11.308	8.218	-3.090	Better	17.173	20.508	3.335	Worse	52%	150%
				(2.672)				(2.813)			
Childhood fever prevalence	*(childfev)*	20.696	20.674	-0.022	Better	25.023	29.206	4.183	Worse	21%	41%
				(3.109)				(3.038)			
Childhood diarrhea prevalence	*(childdiarr)*	19.250	15.168	-4.082	Better	13.931	20.003	6.072***	Worse	-28%	32%
				(3.112)				(2.344)			
Under-5 mortality	*(childmort)*	3.537	2.717	-0.819	Better	2.261	3.930	1.669	Worse	-36%	45%
				(1.126)				(1.165)			

Standard errors are reported in parentheses. *, **, and *** denote statistical significance at the 10%, 5%, and 1% levels, respectively

Most health outcomes appear to have steadily improved over time for the two groups of facilities. On average, seven out of ten outcomes improved between 2005 and 2014 for non-contracted facilities. Five outcomes improved for contracted facilities over the same period. However, the magnitudes of the improvements for contracted facilities are smaller than for non-contracted facilities for all outcomes, excluding modern contraceptive prevalence *(mcp).* For instance, between 2005 and 2014, ANC by skilled health personnel *(ancprov)* and coverage of at least four ANC visits *(anc4)* increased by 17 ppts and 27 ppts, respectively, for contracted facilities. These increases are far less than the increases reported for non-contracted facilities for the two outcomes (28 ppts and 40 ppts, respectively)

Table 4 also shows that the child health outcomes of contracted facilities deteriorated between 2005 and 2014 (prevalence of childhood ARI *(childari)*, 150 ppts; under-5 mortality *(childmort)*, 45 ppts; prevalence of childhood fever *(childfev)*, 41 ppts; prevalence of childhood diarrhea *(childdiarr)*, 32 ppts). In parallel, two child health outcomes improved for non-contracted facilities (under-5 mortality *(childmort)*, -36 ppts; prevalence of childhood diarrhea *(childdiarr)*, -28 ppts). Contracted facilities appear to be significantly worse off concerning the prevalence of childhood diarrhea *(childdiarr)* post the PBF discontinuation (as observed in 2014).

### Estimated effects of discontinuing provider incentives

Tables 5, 6, and 7 present the DiD fixed-effects estimates of the effects of contracting and, importantly, discontinuing PBF of healthcare providers at contracted PHC facilities on family planning, maternal health, and child health outcomes over the 1992–2014 period. Overall, the reported estimates indicate that PBF discontinuation negatively affected some of the health outcomes of contracted facilities, but the observed negative effects are significant *only* for indirectly targeted (non-contracted) outcomes.

For family planning, Table 5 shows a significant negative effect on the knowledge of contraceptive methods. The percentage of women with access to contracted facilities, who were informed of alternative contraceptives *(contoth)*, decreased by about 11.3 ppts due to the PBF discontinuation. This negative effect surpasses the positive effect that contracting initially had on *contoth*, in terms of magnitude and significance.

As stated earlier, one of the indicators upon which a contracted facility qualified for financial incentives was the protection provided by family planning services, denoted by the years of protection. But we find that discontinuing the financial incentives had no effect on modern contraceptive prevalence *(mcp)* among women with access to contracted facilities. We argue that the performance-based incentives, once in operation, managed to create beneficial habits among providers at contracted facilities, which were transferred into a permanent change in women’s contraceptive behavior. That is, those women with access to contracted facilities now permanently uptake modern contraception. In Egypt, it seems that this was the case for the practice of contraception *(mcp)* but not the knowledge of contraceptives *(contoth)*.

**Table 5:** Estimated *selectivity-robust* effects of PBF discontinuation on family planning, 1992–2014

	Modern contraceptive prevalence	Knowledge of side effects of contraceptives	Knowledge of alternative contraceptives
	*(mcp)*	*(contsid)*	*(contoth)*
Contracting = 1	1.203	3.502	9.221**
	(2.488)	(4.208)	(4.349)
PBF discontinuation = 1	1.919	0.928	-11.329**
	(2.851)	(4.725)	(4.846)
Years (Ref: 1992)			
1995	0.537	-3.394	
	(1.548)	(2.929)	
2000	5.284***	35.648***	41.416***
	(1.560)	(2.634)	(2.344)
2005	11.106***	35.550***	45.458***
	(1.502)	(2.506)	(2.196)
2008	10.911***	39.821***	50.692***
	(1.553)	(2.604)	(2.312)
2014	9.124***	36.207***	50.829***
	(1.553)	(2.594)	(2.313)
Constant	38.792***	8.647***	5.661***
	(1.208)	(2.028)	(1.614)
Obs.	2,966	2,683	2,624

Each *column* represents a separate regression. Standard errors are reported in parentheses. *, **, and *** denote statistical significance at the 10%, 5%, and 1% levels, respectively. Facility and year fixed effects are included in all estimations

Regarding ANC, Table 6 reveals that discontinuing performance-based incentives had no effect on the two relevant directly targeted (contracted) outcomes: the likelihood of receiving ANC by skilled health personnel *(ancprov)* and ANC coverage (at least four visits) *(anc4)*. A significant negative effect is reported for the likelihood of receiving iron supplements during pregnancy *(anciron)*, which is a non-contracted outcome. The percentage of women with access to contracted facilities, who received iron supplements during pregnancy *(anciron)*, decreased by about 9.4 ppts following the PBF discontinuation.

**Table 6:** Estimated *selectivity-robust* effects of PBF discontinuation on ANC, 1992–2014

	ANC by skilled health personnel	4 + visits	Iron supplementation
	*(ancprov)*	*(anc4)*	*(anciron)*
Contracting = 1	2.844	-1.968	6.705
	(3.320)	(3.452)	(4.385)
PBF discontinuation = 1	1.676	2.972	-9.448**
	(3.809)	(3.960)	(4.641)
Years (Ref: 1992)			
1995	-16.699***	4.933**	
	(2.082)	(2.164)	
2000	-0.850	16.203***	
	(2.097)	(2.181)	
2005	16.028***	38.745***	26.303***
	(2.012)	(2.091)	(2.074)
2008	15.044***	40.078***	10.363***
	(2.081)	(2.164)	(2.189)
2014	28.484***	54.473***	38.562***
	(2.085)	(2.170)	(2.169)
Constant	56.619***	23.816***	26.812***
	(1.621)	(1.686)	(1.455)
Obs.	2,947	2,946	2,250

Each *column* represents a separate regression. Standard errors are reported in parentheses. *, **, and *** denote statistical significance at the 10%, 5%, and 1% levels, respectively. Facility and year fixed effects are included in all estimations

Note that both *ancprov* and *anc4* were directly and strongly related to provider incentives in the first instance. The choice of a woman to use the skilled care of a healthcare provider is influenced by the quality of care the provider offers. In this sense, if discontinuing the financial incentives demotivated providers from maintaining the high quality of care, women could become less prone to attend ANC run by skilled health personnel. Interestingly, the estimates in Table 6 show *no* evidence of this effect for contracted facilities, again suggesting that public health priority areas *directly* targeted by the PBF scheme were not likely to deteriorate after the scheme discontinuation.

However, considering child health outcomes *indirectly* targeted by the PBF scheme through *intermediate* indicators, such as the number of visits per day per physician or even the rate of patient referral to the district hospital, we detect significant negative effects for contracted facilities (Table 7). The same applies to the quality of ANC provided in contracted facilities, reflected by iron supplementation during pregnancy *(anciron)*, versus the volume of ANC service utilization *(anc4)* (Table 6). These findings warrant further investigation into why non-contracted health outcomes are more likely to deteriorate post PBF discontinuation than contracted ones.

**Table 7:** Estimated *selectivity-robust* effects of PBF discontinuation on child health, 1992–2014

	ARI prevalence	Fever prevalence	Diarrhea prevalence	Under-5 mortality
	*(childari)*	*(childfev)*	*(childdiarr)*	*(childmort)*
Contracting = 1	-5.877**	-2.988	-2.147	-1.231
	(2.468)	(3.016)	(2.278)	(1.149)
PBF discontinuation = 1	5.494*	1.583	1.327	2.680**
	(2.843)	(3.473)	(2.624)	(1.318)
Years (Ref: 1992)				
1995	13.906***	20.096***	1.756	0.046
	(1.546)	(1.889)	(1.427)	(0.721)
2000	0.876	-3.843**	-6.463***	-1.965***
	(1.558)	(1.904)	(1.438)	(0.726)
2005	2.899*	-0.249	3.520**	-1.846***
	(1.494)	(1.826)	(1.379)	(0.696)
2008	2.617*	-7.694***	-4.786***	-2.573***
	(1.546)	(1.889)	(1.427)	(0.721)
2014	6.875***	1.850	-0.748	-3.548***
	(1.548)	(1.892)	(1.429)	(0.722)
Constant	9.131***	22.313***	14.666***	5.842***
	(1.204)	(1.471)	(1.111)	(0.561)
Obs.	2,944	2,944	2,944	2,947

Each *column* represents a separate regression. Standard errors are reported in parentheses. *, **, and *** denote statistical significance at the 10%, 5%, and 1% levels, respectively. Facility and year fixed effects are included in all estimations

The most worrying effects are observed for child health, for which the point estimate in Table 7 is positive for all four outcomes, reflecting increased child morbidity and mortality. The discontinuation of the PBF scheme significantly increased the prevalence of childhood ARI *(childari)* among children with access to contracted facilities by about 5.5 ppts. The scheme discontinuation also had a significant negative effect on child mortality *(childmort)*, which increased by about 2.7 ppts among children with access to contracted facilities after the PBF scheme became no longer in operation. The observed negative effects strongly suggest that replacing performance-based incentives with case-based reimbursement led to lower quality of child health services, which deterred women from using them. Such effects can be exacerbated by the rising frequency of health disasters worldwide and the significant indirect impact of these disasters on maternal and child mortality in LMICs (El-Shal et al., 2022).

## Conclusions

This study provides novel evidence on the effect of *discontinuing* performance-based provider incentives in PHC in LMICs on the health services targeted by the PBF scheme and on non-contracted outcomes. A unique panel dataset of the health outcomes of PHC facilities across Egypt is *spatially* constructed based on data from six waves of the Egypt DHS for the period 1992–2014. Building on the parallel-trend assumption, we employ a DiD model with fixed effects.

Regarding family planning, we find that discontinuing performance-based incentives had a significant adverse effect on the knowledge of alternative contraceptive methods (a decrease of 11.3 ppts) but no effect on the knowledge of side effects of contraceptives and, notably, modern contraceptive prevalence. These results suggest that healthcare providers and women with access to contracted facilities acquired favorable habits during the PBF operation, even if temporarily. It appears that these changes in habits among women with access to those facilities became permanent in their contraceptive behavior.

As for ANC, discontinuing the incentives had a significant negative effect on the likelihood of receiving iron supplements during pregnancy (-9.4 ppts), a *non-contracted* outcome. However, there was no effect on *contracted* outcomes, namely the likelihoods of receiving ANC by skilled health personnel and of receiving four or more ANC visits.

The most worrying effects of the incentives’ discontinuation are observed for child morbidity and mortality. The discontinuation of the PBF scheme significantly increased the prevalence of childhood ARI and, more importantly, under-five mortality among children with access to contracted facilities by 5.5 ppts and 2.7 ppts, respectively. The negative effects on these *non-contracted* outcomes indicate that replacing performance-based incentives with case-based reimbursement led to a lower quality of child health services, which might have deterred women from using them.

Our findings suggest that performance-based incentives can create habits, e.g., permanent adoption of the modified behavior by healthcare providers with possible spillover effects on women in the catchment areas. However, PBF schemes need to be applied carefully in LMICs as negative effects can be observed upon their discontinuation for key but non-contracted health outcomes, such as child mortality (the case of Egypt).

## Data Availability

Subject to approvals by The DHS Program and Egypt’s Ministry of Health and Population.

## Appendix A

**Existing evidence on introducing provider incentives**

We additionally reviewed the evidence on the effects of introducing provider incentives in an attempt to anticipate the likely effects of discontinuing these incentives in LMICs.

Four systematic reviews of the literature were relevant for the scope of this study: James et al. (2020), Das et al. (2016), Witter et al. (2012), and Eldridge and Palmer (2009). Reviewing the evidence on the effect of RBF schemes on maternal, newborn, and child health in LMICs, James et al. (2020) indicated significant positive effects on maternal and child health coverage indicators and structural measures of the quality of care in most covered LMICs.

Das et al. (2016) assessed the existing evidence on the effects of PBF on the quality of maternal and child health care in LMICs. The review found some evidence that PBF was associated with positive effects but only on maternal and child healthcare process quality. This included adherence to standard protocols and guidelines for the management of health conditions. The effects of PBF on delivery, emergency obstetric and neonatal care, postnatal care, and under-five child care were not investigated in the studies included in this review. Importantly, Das et al. (2016) found weak evidence that PBF was associated with positive effects on maternal and neonatal health outcomes and OOP expenses. PBF was also found to have a few negative effects on structural quality.

Witter et al. (2012) assessed the existing evidence on the effects of PBF on the provision of health care and health outcomes in LMICs. The review concluded that the current evidence is too weak to draw general conclusions. The evidence provided suggests that the effects of PBF depend on the interaction of several variables. A key variable is the design of the PBF scheme (e.g., who receives payments, the magnitude of the incentives, the targets and how they are measured). Other key variables include but are not limited to the amount of funding the PBF scheme receives, the strength of technical support PBF gets, and contextual factors, such as the organizational context in which PBF is implemented.

Eldridge & Palmer (2009) assessed the existing evidence on PBF in low-income countries. Their review spotted significant weakness in the existing evidence on the effectiveness of PBF initiatives. They concluded that the lack of evidence on the effects of any type of PBF in low-income countries is primarily due to the absence of control groups.

Our own systematic review of studies that explore the effects of introducing provider incentives in LMIC settings suggests that the available evidence is mixed and of low quality. The evidence is particularly limited when investigating the effects on patient outcomes rather than process indicators. Our review focused on (1) family planning, (2) maternal health, and (3) child health outcomes.

**Effect on family planning.** The effects of PBF on family planning outcomes are limited. Four of the eight studies reviewed reported no effects (Zizien et al., 2019; Engineer et al., 2016; Falisse et al., 2015; Soeters et al., 2011). Soeters et al. (2011) determined that PBF did not significantly affect family planning output and patient knowledge indicators in the Democratic Republic of Congo, despite individuals having heard about family planning and currently using a modern contraceptive method. Even when accompanied by removing user fees, Falisse et al. (2015) found no effect of PBF in Burundi on fittings of intra-uterine devices (IUD) as a family planning method. Similarly, no significant effect on modern contraception was reported in Burkina Faso and Afghanistan (Zizien et al., 2019; Cyrus et al., 2015). However, multiple interventions in Egypt, including PBF, were associated with increases in the use of modern contraceptive methods (Grun & Ayala, 2006). Besides PBF, interventions in Egypt included quality improvement through facility accreditation and user fee introduction. PBF was associated with significant increases in the share of women using modern family planning services in Cameroon and Burundi (Walque et al., 2017; Bonfrer et al., 2014a; Rajkotia et al., 2017) also demonstrated that PBF positively affected family planning consultations for HIV-infected women in Mozambique.

**Effect on maternal health.** A total of 15 studies reported on the effects of PBF on maternal health. The obtained evidence suggests that PBF had variable effects on maternal health.

Out of the 15 studies, 13 reported ANC measured by the number of visits. Five studies reported positive effects of PBF on the number of ANC visits in Mozambique, Burkina Faso, Burundi, Argentina, and Bangladesh (Rajkotia et al., 2017; Steenland et al., 2017; Falisse et al., 2015; Gertler et al., 2014; Nguyen et al., 2012). The latter three studies did not investigate the effect of PBF as a stand-alone policy. In all three studies, PBF was introduced together with other demand-side interventions that involved removing the cost of care. The remaining eight studies reporting on ANC found no effect on the number of ANC visits in Burkina Faso, Cameroon, Burundi (two studies), Rwanda, Afghanistan, Cambodia, and Egypt, respectively (Zizien et al., 2019; Walque et al., 2017; Rudasingwa et al., 2017; Bonfrer et al., 2014b; Van de Poel et al., 2016; Cyrus et al., 2015; Basinga et al., 2011; Grun & Ayala, 2006).

Despite the limited effect of PBF on the number of ANC visits, all five studies reporting on the quality of ANC found a positive effect of PBF on the quality of ANC. Walque et al. (2017) indicated significant improvements in Cameroon’s structural quality of care as measured by equipment availability, staff presence, and staff satisfaction. In Rwanda, PBF significantly improved the quality of care measured by anti-tetanus vaccination during ANC and standardized total quality scores. However, this improvement was not associated with an increase in the use of care measured by the probability that women receive any ANC or that they have at least four ANC visits (Basinga et al., 2011). Similar effects were captured by Bonfrer et al. (2014b) in Burundi, where the quality of care provided during ANC visits improved significantly due to PBF even though the number and timeliness of visits did not change. The quality of ANC was captured by both blood pressure (BP) measurement and anti-tetanus vaccination. Falisse et al. (2015) and Gertler et al. (2014) also reported a significant positive effect of PBF in Burundi and Argentina, respectively, on anti-tetanus vaccination.

Six out of the ten studies reporting on institutional delivery found significant positive effects on delivery care. Steenland et al. (2017) reported a significant increase in the number of complicated and uncomplicated deliveries at PBF health facilities in Burkina Faso. In Rwanda, Basinga et al. (2011) captured a significant increase in the probability of institutional delivery associated with PBF. The PBF scheme in the Democratic Republic of Congo was also associated with a significant increase in institutional delivery (Soeters et al., 2011). Similarly, Van de Poel et al. (2016) captured a significant increase in the percentage of births occurring in incentivized public health facilities in Cambodia. However, the effect on delivery in public facilities was greater if PBF was accompanied by maternity vouchers covering user fees. No significant effects were observed among the poorest women. Nguyen et al. (2012) provided evidence that combining cash incentives for individuals (i.e., vouchers) with cash incentives for healthcare providers could significantly increase institutional delivery. Walque et al. (2017) indicated no effect of PBF on institutional delivery in Cameroon. Both Falisse et al. (2015) and Bonfrer et al. (2014b) indicated no effect in Burundi. But Rudasingwa et al. (2017) reported a positive effect for the latter. Inconclusive evidence was provided by Rajkotia et al. (2017) in Mozambique.

No effect of PBF was detected on skilled-assisted delivery and postnatal care in Cameroon and Afghanistan (Walque et al., 2017; Cyrus et al., 2015), but positive effects were reported in Burkina Faso (two studies) and Mozambique on postnatal consultation coverage (Zizien et al., 2019; Steenland et al., 2017; Rajkotia et al., 2017).

Importantly, a recent study by De Allegri et al. (2019) shows that RBF significantly reduced facility-based maternal mortality at birth in Malawi.

**Effect on child health.** A total of 13 studies reported on the effects of PBF on child health. The identified studies provide evidence that PBF generally had limited effects on child health outcomes.

A total of 11 out of the 13 studies on child health reported on the effects of vaccination, with only three studies out of the 11 capturing significant positive effects of PBF. Walque et al. (2017) indicated a significant positive effect of PBF on full vaccination coverage of children under five years of age in Cameroon. Bonfrer et al. (2014b) found PBF significantly increased the probability of a child being fully vaccinated in Burundi, with the effects being more pronounced among the poor. In Egypt, PBF accompanied by other interventions significantly increased the measles vaccination rate (Grun & Ayala, 2006). However, no effect of PBF on vaccination rates was observed in Burkina Faso, Mozambique, Cambodia, Afghanistan, Burundi, Argentina, Rwanda, and the Democratic Republic of Congo (Zizien et al., 2019; Rajkotia et al., 2017; Van de Poel et al., 2016; Cyrus et al., 2015; Falisse et al., 2015; Gertler et al., 2014; Basinga et al., 2011; Soeters et al., 2011; respectively).

Evidence on child mortality is limited. Only three studies reported on the effects of PBF on child mortality, one out of which captured significant positive effects in Argentina (Gertler et al., 2014). The study showed that combining health insurance with PBF had significant positive effects on child health, as beneficiaries had a lower chance of in-hospital neonatal mortality. Approximately half of this reduction resulted from preventing low birth weight and a half from better postnatal care. In parallel, Van de Poel et al. (2016) and Falisse et al. (2015) did not find any effects of PBF on neonatal mortality and perinatal mortality, respectively, in Cambodia and Burundi.

For medical treatment, Skiles et al. (2015) reported a significant positive effect of PBF on treatment received by children with diarrhea or fever at health facilities in Rwanda. Similarly, multiple interventions in Egypt, including PBF, significantly improved child access to medical treatment (fever/cough). However, there was no effect on the share of diarrhea cases in children receiving medical treatment (Grun & Ayala, 2006).

PBF was not associated with adverse effects on non-incentivized indicators in Mozambique (Rajkotia et al., 2017). But in Tanzania, a government pay-for-performance scheme had a negative effect on non-targeted general outpatient service use and a limited positive effect on the utilization of maternal and child immunization services targeted by the scheme (Binyaruka et al., 2015).

**Summary.** Overall, the evidence on the effectiveness of introducing PBF on improving family planning, maternal health, and child health outcomes is mixed. This is partly because PBF schemes introduced in LMICs are not identical. The most consistent evidence of the effectiveness of PBF is for the quality of ANC and medical treatment among children. These are the areas that we expect to be affected the most if performance-based incentives to healthcare providers are discontinued.

## Appendix B

**Description of the Egypt DHS**.

**Table:**

	The 1992 Egypt DHS	The 1995 Egypt DHS	The 2000 Egypt DHS	The 2005 Egypt DHS	The 2008 Egypt DHS	The 2014 Egypt DHS
Sample selection						
Stage 1	378 primary sampling units selected (169 shiakhas/towns and 209 villages)	467 primary sampling units selected (204 shiakhas/towns and 263 villages)	500 primary sampling units selected (228 shiakhas/towns and 272 villages)	682 primary sampling units selected (298 shiakhas/towns and 384 villages)	610 primary sampling units selected (275 shiakhas/towns and 335 villages)	884 primary sampling units selected (481 shiakhas/towns and 445 villages before dropping North and South Sinai from the sample)
Stage 2	546 segments from the parts in each shiakha/town and village chosen	934 segments from the parts in each shiakha/town and village chosen	1,000 segments from the parts in each shiakha/town and village chosen	1,359 segments from the parts in each shiakha/town and village chosen	1,267 segments from the parts in each shiakha/town and village chosen	1,838 segments from the parts in each shiakha/town and village chosen
Stage 3	Random sample of HHs drawn	Random sample of HHs drawn	Random sample of HHs drawn	Random sample of HHs drawn	Random sample of HHs drawn	Random sample of HHs drawn
Survey coverage	11,304 HHs	16,046 HHs	17,521 HHs	22,807 HHs	19,739 HHs	29,471 HHs
	9,978 women	14,879 women	15,649 women	19,565 women	16,571 women	21,903 women
Response rate	98.9%	99.3%	99.5%	99.5%	99.7%	99.4%

*Source*: The Egypt DHS, 1992, 1995, 2000, 2005, 2008, and 2014

## Footnotes

1 Under the English fundholding scheme, general practices could elect to be given a budget to meet the costs of certain types of elective surgery for their patients and could retain any surplus.

2 The HIO is both a financer and provider of care.

3 The FHFs were initially established at the governorate level in 1999 to separate the financing from the provision of PHC services and to ensure the sustainability of finance of PHC services. The FHFs acted as intermediate contractors that purchased PHC services from healthcare providers (public, private, and NGOs) on behalf of uninsured and insured beneficiaries. The FHFs established and supervised the rules and eligibility criteria for PHC providers, contracted with providers provided accreditation and adherence to the FHFs’ guidelines, rechanneled the funds allocated to PHC services by receiving finance from a mix of sources, and paid salaries of staff.

4 The index was constructed based on eight social and economic indicators covering illiteracy, unemployment, income dependency, inaccessibility to electricity, inaccessibility to potable water, family size, household (HH) overcrowding, and population size.

5 It should be noted that while designing incentive-based contracts for healthcare providers (agents), it is important for the purchaser of services (the principal) to seek to maximize its utility subject to the individual rationality and incentive compatibility constraints of the provider (the agent). This is a typical moral hazard situation.

## Code availability

Upon request.
